# Effectiveness of Stereotactic Ablative Radiotherapy for Systemic Therapy Respondents with Inoperable Pulmonary Oligometastases and Oligoprogression

**DOI:** 10.3390/diagnostics13091597

**Published:** 2023-04-29

**Authors:** Chin-Beng Ho, Jo-Ting Tsai, Chun-You Chen, Her-Shyong Shiah, Hsuan-Yu Chen, Lai-Lei Ting, Chia-Chun Kuo, I-Chun Lai, Hsin-Yi Lai, Chi-Li Chung, Kai-Ling Lee, Huey-En Tzeng, Kuen-Haur Lee, Hsin-Lun Lee, Shang-Wen Chen, Jeng-Fong Chiou

**Affiliations:** 1 Ph.D. Program for Cancer Molecular Biology and Drug Discovery, College of Medical Science and Technology, Taipei Medical University and Academia Sinica, Taipei Medical University, Taipei 110301, Taiwan; ellenisho@gmail.com (C.-B.H.);; 2 Department of Radiation Oncology, Camillian Saint Mary’s Hospital Luodong, Yilan 265502, Taiwan; 3 Department of Radiation Oncology, Taipei Medical University Hospital, Taipei 110301, Taiwan; 4 Department of Radiation Oncology, Taipei Medical University-Shuang Ho Hospital, New Taipei City 235041, Taiwan; 5 Department of Radiology, School of Medicine, College of Medicine, Taipei Medical University, Taipei 110301, Taiwan; 6 Taipei Cancer Center, Taipei Medical University, Taipei 110301, Taiwan; 7 Department of Radiation Oncology, Wan Fang Hospital, Taipei Medical University, Taipei 116079, Taiwan; 8 Division of Hematology and Oncology, Department of Internal Medicine, Taipei Tzu Chi Hospital, Buddhist Tzu Chi Medical Foundation, New Taipei City 231016, Taiwan; 9 Graduate Institute of Cancer Biology and Drug Discovery, College of Medical Science and Technology, Taipei Medical University, Taipei 110301, Taiwan; 10 Institute of Statistical Science, Academia Sinica, Taipei 115201, Taiwan; 11 Department of Heavy Particles and Radiation Oncology, Taipei Veterans General Hospital, Taipei 112201, Taiwan; 12 School of Medicine, National Yang Ming Chiao Tung University, Taipei 112304, Taiwan; 13 Department of Medical Imaging, Taipei Medical University Hospital, Taipei Medical University, Taipei 110301, Taiwan; 14 Division of Pulmonary Medicine, Department of Internal Medicine, Taipei Medical University Hospital, Taipei Medical University, Taipei 110301, Taiwan; 15 School of Respiratory Therapy, College of Medicine, Taipei Medical University, Taipei 110301, Taiwan; 16 Division of Thoracic Medicine, Department of Internal Medicine, School of Medicine, College of Medicine, Taipei Medical University, Taipei 110301, Taiwan; 17 Division of Hematology/Medical Oncology, Department of Medicine, Taichung Veterans General Hospital, Taichung 407219, Taiwan; 18 Department of Radiation Oncology, China Medical University Hospital, Taichung 404327, Taiwan; 19 Graduate Institute of Biomedical Sciences, School of Medicine, College of Medicine, China Medical University, Taichung 404333, Taiwan

**Keywords:** pulmonary metastases, oligometastases, oligoprogression, extrapulmonary disease, metastasectomy, stereotactic ablative radiotherapy (SABR)

## Abstract

Stereotactic ablative radiotherapy (SABR) may improve survival in patients with inoperable pulmonary oligometastases. However, the impact of pulmonary oligometastatic status after systemic therapy on SABR outcomes remains unclear. Hence, we investigated the outcomes of SABR in 45 patients with 77 lung tumors and the prognostic value of pulmonary oligoprogression. Eligibility criteria were pulmonary oligometastases (defined as ≤5 metastatic lung tumors), controlled extrapulmonary disease (EPD) after front-line systemic therapy, SABR as primary local treatment for inoperable pulmonary metastases, and consecutive imaging follow-up. Oligometastatic lung tumor was classified into controlled or oligoprogressive status. Overall survival (OS), in-field progression-free survival (IFPFS), out-field progression-free survival (OFPFS), and prognostic variables were evaluated. With 21.8 months median follow-up, the median OS, IFPFS, and OFPFS were 28.3, not reached, and 6.5 months, respectively. Two-year OS, IFPFS, and OFPFS rates were 56.0%, 74.2%, and 17.3%, respectively. Oligoprogressive status (*p* = 0.003), disease-free interval < 24 months (*p* = 0.041), and biologically effective dose (BED_10_) < 100 Gy (*p* = 0.006) were independently associated with inferior OS. BED_10_ ≥ 100 Gy (*p* = 0.029) was independently correlated with longer IFPFS. Oligoprogressive status (*p* = 0.017) and EPD (*p* = 0.019) were significantly associated with inferior OFPFS. Grade ≥ 2 radiation pneumonitis occurred in four (8.9%) patients. Conclusively, SABR with BED_10_ ≥ 100 Gy could provide substantial in-field tumor control and longer OS for systemic therapy respondents with inoperable pulmonary oligometastases. Oligoprogressive lung tumors exhibited a higher risk of out-field treatment failure and shorter OS. Hence, systemic therapy should be tailored for patients with oligoprogression to reduce the risk of out-field treatment failure. However, in the absence of effective systemic therapy, SABR is a reasonable alternative to reduce resistant tumor burden.

## 1. Introduction

Neoplasm metastasis is a major cause of cancer-related deaths, and its treatment outcomes remain unsatisfactory despite recent advances in systemic therapy, which for decades has been the standard treatment for metastatic diseases [1,2,3]. Nevertheless, Hellman et al. [4] proposed the concept of oligometastases (≤5 metastatic lesions), an intermediate state between limited primary and widespread disease in which a metastasectomy may contribute to survival in selected patients [5]. More recently, Niibe et al. [6,7] defined an oligometastatic state with a controlled primary tumor as “oligo-recurrence”, which confers a more favorable prognosis than polymetastases. For oligo-recurrence, the integration of local ablative therapies such as surgical metastasectomy, stereotactic ablative radiotherapy (SABR), radiofrequency ablation, and cryoablation is recommended [6,7].

Pulmonary metastasis is highly prevalent across various cancer types and results in considerable cancer morbidity and mortality [8,9,10]. Pulmonary metastasectomy has been demonstrated to prolong survival and is considered the first-line local treatment for patients with pulmonary oligometastases [11,12,13], whereas SABR has been reported to result in substantial tumor control with acceptable treatment-related toxicities and serves as an alternative local treatment for patients deemed inoperable. [14,15,16] In such cases, favorable treatment outcomes have been observed in patients with controlled primary tumor [17,18,19,20]. With recent advances in systemic therapy, the phenomenon of oligoprogression is increasingly recognized as the contradictory progression of a limited metastatic lesion despite the disease being controlled elsewhere, indicating divergent resistance in response to systemic treatment for metastatic tumors. Such tumors may also benefit from local ablative treatment [21,22,23].

Oligoprogression exhibits distinct biological signatures during the evolution of cancer metastasis. However, its clinical significance has not been clarified. We conducted this retrospective study to investigate the treatment outcomes of SABR in systemic therapy respondents with inoperable pulmonary oligometastases and analyze the prognostic significance of oligoprogressive versus controlled status of metastatic lung tumors.

## 2. Materials and Methods 

### 2.1. Study Design, Patients, and Treatments

**Figure 1:**
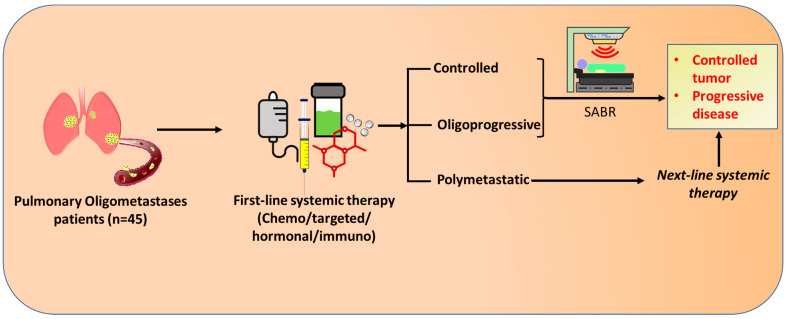
Schematic design of the study. The patients with inoperable pulmonary oligometastases at diagnosis had been receiving first-line systemic therapy consisting of chemotherapy, targeted therapy, hormonal therapy, and/or immunotherapies. Upon radiographic re-examination for treatment response, if the pulmonary oligometastases continued to be inoperable and without evidence of polymetastases (Controlled or oligoprogressive), SABR was administered. However, the patients in a polymetastatic state were treated with next-line systemic therapy, which further lead to either controlled or progressive disease. SABR: Stereotactic Ablative radiotherapy.

We retrospectively reviewed the medical records of patients with pulmonary metastases treated with SABR at Taipei Medical University Hospital and China Medical University Hospital, Taiwan between June 2009 and June 2019. The study design (Figure 1) was approved by the Taipei Medical University-Joint Institutional Review Board (TMU-JIRB No. N202105033). Inclusion criteria for patient enrollment were (1) the presence of pulmonary oligometastases with controlled primary tumor and extrapulmonary disease (EPD) after standard front-line systemic therapy for at least 3 months, (2) SABR as the primary local treatment for oligometastatic lung tumors, and (3) a consecutive imaging follow-up of 4 months. In our institutes, an appropriate therapeutic strategy to manage pulmonary oligometastases was determined by a tumor board with a multidisciplinary cancer team. 

Pulmonary metastasectomy has been recommended as the first-line local treatment for oligometastases patients with controlled primary tumors and EPD. SABR was reserved for those who had a medically or surgically inoperable condition or refused surgery and had a fair performance status, as determined by an Eastern Cooperative Oncology Group score of ≤2. Pulmonary oligometastases were defined as ≤5 metastatic lung tumors with absent or controlled EPD. Metastatic lung tumors were classified into controlled or oligoprogressive status according to their treatment response to systemic therapy at 3 months or longer treatment duration. Oligoprogressive status was a phenomenon of contradictory progression of oligometastatic lung tumors despite EPD responding to systemic therapy. Controlled status was defined as stable disease or partial remission of oligometastatic lung tumors after systemic therapy. Further, the metastatic characteristics were also identified for the entire patient cohort. Synchronous metastases were considered those diagnosed simultaneously (≤3 months) with primary cancer, whereas metachronous metastases were defined as those occurring after at least a four-month interval following the primary cancer diagnosis [24]. The disease-free interval (DFI) between primary cancer treatment and the onset of initial metastases was determined for each patient [18]. 

### 2.2. Stereotactic Ablation Radiotherapy (SABR) 

During computed tomography (CT) simulation and treatment, patients were immobilized using a vacuum cushion and body immobilization device, and subsequently forced to undergo shallow breathing with an abdominal compressor. Four-dimensional CT images with a slice thickness of 3 mm were acquired after intravenous contrast injection. Target lesions or gross tumor volumes were identified using the CT lung window setting. The internal tumor volume (ITV) was delineated using a maximum-intensity projection image, which constituted the maximum target movement during free breathing. The planning target volume (PTV) was designed according to an isotropic 3–6 mm expansion of the ITV, which accounted for various setup errors according to the International Commission on Radiation Units Report 62 [25]. The dosimetry treatment plans were calculated with TomoTherapy (TomoTherapy Inc., Madison, WI, USA), Pinnacle (Philips Medical Systems, Inc., Milpitas, CA, USA), or Eclipse version 6.2 or 8.1 (Varian Medical Systems Inc., Palo Alto, CA, USA). SABR was delivered using 6-MV photon image-guided RT with a TomoTherapy accelerator (Accuray Inc., Sunnyvale, CA, USA), Elekta Synergy linear accelerator (Elekta AB, Stockholm, Sweden), or Varian Clinac iX linear accelerator (Varian Medical Systems Inc.) equipped with an online cone-beam CT device. The total prescribed dose ranged from 30–60 Gy in 3–5 fractions and was adapted to adjacent normal organ constraints and tumor location. The normal tissue constraints are listed in Supplementary Materials S1. The PTV was covered by a ≥95% prescribed isodose, which was normalized to the maximum dose. To correlate various fractionation schedules with treatment efficacy, a biologically effective dose (BED) based on a linear-quadratic model was utilized [26]. The BED_10_ was calculated as *nd* [1 + *d*/(*α*/*β*)], where *n* and *d* represent the number of fractions and fraction size, respectively, and an *α*/*β* ratio of 10 Gy was assumed for metastatic lung tumors [27]. 

### 2.3. Data Analysis

The primary endpoint was overall survival (OS), whereas the secondary endpoints were in-field progression-free survival (IFPFS) and out-field progression-free survival (OFPFS). Treatment responses were measured using the Response Evaluation Criteria in Solid Tumors guidelines (version 1.1). Treatment-related toxicity was scored according to the Common Terminology Criteria for Adverse Events (version 4.03). The OS was defined as the time from the SABR commencement date to the date of last follow-up or death. IFPFS and OFPFS were defined as the time from the SABR commencement date to the date of radiological progression within the irradiated field or at the margin and outside the irradiated field, respectively. Contrast-enhanced CT was employed to assess lung tumors. EPD was evaluated using CT, magnetic resonance imaging, bone scintigraphy, or positron emission tomography for different sites. OS, IFPFS, and OFPFS were calculated using the Kaplan–Meier analysis, and a log-rank test was used for group comparisons. Cox proportional hazard regression was performed to examine the effects of variables. Variables with a *p*-value of <0.10 in the univariate analysis were considered potential prognostic factors and included in the multivariate analysis. A stepwise variable selection procedure was implemented. During the backward selection procedure, we excluded the variable with the highest *p* values > 0.10. All statistical analyses were performed using SPSS version 23 (IBM, Armonk, New York, NY, USA), and *p* values of <0.05 were considered statistically significant. 

## 3. Results

### 3.1. Patient, Tumor Characteristics, and Radiation Regimens

We identified a total of 165 pulmonary oligometastases patients treated with SABR. Of these, 45 met the inclusion criteria and were enrolled for analyses, the tumor and treatment characteristics of whom are given in Table 1. Our cohort identified 12 primary cancer types with hepatocellular carcinoma (HCC) (35.6%) and colorectal cancer (28.9%) being the most common. Oligoprogressive lung tumors were found in 16 (35.6%) patients. Thirty (66.7%) patients received subsequent systemic therapy. Of these, 19, 10, and 1 patient underwent chemotherapy, targeted therapy, and immunotherapy, respectively. A median BED_10_ of 100 Gy (range: 59.5–132.0 Gy) with a median fraction number of 5 (range: 3–6) was prescribed to treat 77 lung tumors.

### 3.2. Treatment Outcomes

**Figure 2:**
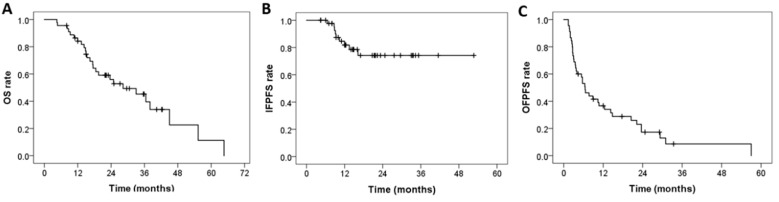
Kaplan–Meier curves of (**A**) OS, (**B**) IFPFS, and (**C**) OFPFS for the entire cohort (N = 45). OS: overall survival, IFPFS: In-field progression-free survival, OFPFS: Out-field progression-free survival.

With a follow-up period of 21.8 months, the median OS (Figure 2A), IFPFS (Figure 2B), and OFPFS (Figure 2C) for the entire cohort were 28.3, not reached, and 6.5 months, respectively. The one-year OS, IFPFS, and OFPFS rates were 84.1%, 81.8%, and 36.7%, respectively, whereas the two-year rates were 56.0%, 74.2%, and 17.3%, respectively. 

The median time of response was 2.5 months (range: 1–9.7 months) and the overall treatment response rate for the entire cohort was 73.3%. Complete and partial response was achieved in 17 (37.8%) and 16 (35.6%) patients, respectively. We further conducted a univariate analysis (Table 2) and multivariate analysis (Table 3) of the results for determining the factors affecting OS, IFPFS, and OFPFS. 

**Figure 3:**
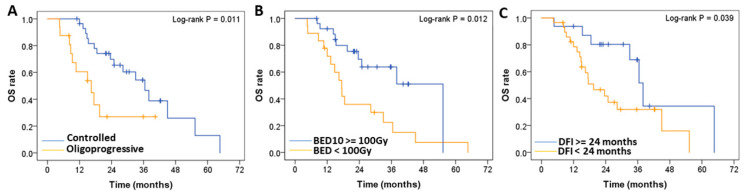
Log-rank test-based comparative analysis of prognostic factors. OS of patients with (**A**) oligoprogressive (N = 16) compared with controlled (N = 29) metastatic lung tumors; (**B**) BED (α/β = 10 Gy), (BED_10_) ≥ 100 Gy (N = 27) compared with BED_10_ < 100 Gy (N = 18); and (**C**) DFI < 24 months (N = 29) compared with DFI ≥ 24 months (N = 16). OS: Overall survival, BED: Biologically effective dose, DFI: disease-free interval.

The independent significant prognostic factors for OS were oligoprogressive status of metastatic lung tumors (hazard ratio [HR] = 4.064; *p* = 0.003) (Figure 3A), BED_10_ ≥ 100 Gy (HR = 0.308; *p* = 0.006) (Figure 3B), and DFI < 24 months (HR = 2.729; *p* = 0.041) (Figure 3C). 

**Figure 4:**
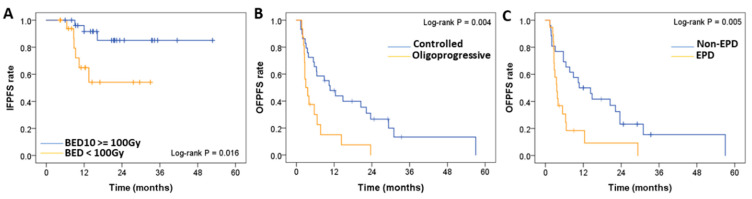
Comparative analysis of (**A**) IFPFS of the patients who received a BED at an α/β ratio of 10 (BED_10_) ≥ 100 Gy (N = 27) and BED_10_ < 100 Gy (N = 18). The OFPFS rate was determined with respect to (**B**) oligoprogressive (N = 16) versus controlled (N = 29) metastatic lung tumors, and (**C**) EPD (N = 19) versus non-EPD. BED: Biologically effective dose, IFPFS: In-field progression-free survival. EPD: Extrapulmonary disease.

The BED_10_ ≥ 100 Gy (HR = 0.211; *p* = 0.029) was the only significant factor for IFPFS (Figure 4A). OFPFS was significantly influenced by the oligoprogressive status of metastatic lung tumors (HR = 2.396; *p* = 0.017) (Figure 4B) and EPD (HR = 2.376; *p* = 0.019) (Figure 4C). The median OFPFS (*p* = 0.004) and OS (*p* = 0.011) for patients with oligoprogressive lung tumors (N = 16) were 3 and 16.4 months, respectively, compared with 10.7 and 36.5 months for patients with controlled metastatic lung tumors (N = 29).

### 3.3. Toxicities

Radiation pneumonitis (RP) ≥ Grade 2 occurred in four (8.9%) patients, one of whom progressed to Grade 3. These patients recovered after conservative treatment. No adverse events over grade 3 were observed during the follow-up period.

## 4. Discussion

This study investigated the treatment outcomes of SABR in patients with inoperable pulmonary oligometastases responding to front-line systemic therapy and the prognostic value of the metastatic lung tumor status. Lung metastases are frequent in many cancer types, such as breast cancer, gastrointestinal malignancies, renal carcinomas, melanoma, and sarcomas owing to blood from the whole body passing through the lungs for gas exchange, which provides a high opportunity for cancer cells seeding in the lungs [28]. The progression of lung metastases leading to respiratory failure is a common etiology of cancer-related death. Hence, it is important to control lung metastases as supported by the many literature studies that demonstrated a survival benefit of metastasectomy and SABR for lung metastases in selected patients [11,12,13,14,15,16].

The primary tumor status has been thoroughly discussed for its influence on overall treatment outcome. In the previous studies, oligometastases in patients with controlled primary tumors, defined as oligo-recurrence, had more favorable treatment outcomes compared with uncontrolled primary disease, as defined as sync-oligometastases [6,7,17,18,19,20,29,30]. However, the prognostic value of oligometastatic tumors remains to be clarified [22,31,32,33,34].

With recent advances in systemic treatment, oligoprogression is increasingly observed in cancer patients with controlled disease elsewhere, indicating the emergence of drug resistance. Local ablative therapies targeting oligoprogressive tumors play a role in improving quality of life and delaying the initiation or modification of systemic therapy [21,35,36]. The biological rationale for treating oligoprogressive tumors with local ablative therapy is that the genetic heterogeneity of primary and metastatic tumors has divergent resistance mechanisms to systemic therapy [37]. Many SABR studies have described clinical outcomes related to oligoprogression [21,22,31,35,38,39,40,41] from non-small-cell lung cancer [21,35,38,39,40], while one study analyzed nodal or bone oligoprogression from prostate cancer [41]. A recent retrospective SABR study revealed that liver oligoprogression may be associated with poor survival [31]. Hence, a growing number of randomized trials have been initiated to clarify the role of SABR for oligometastases and oligoprogression [42]. Our study revealed that the oligoprogressive status of metastatic lung tumors exhibits inferior OS (HR = 4.064; *p* = 0.003) and OFPFS (HR = 2.396; *p* = 0.017) after local ablative therapy using SABR. Nonetheless, adjuvant systemic therapy did not significantly impact OS (*p* = 0.310) and OFPFS (*p* = 0.348), which highlights the dilemma of drug selection.

Conversely, BED_10_ ≥ 100 Gy (HR = 0.308; *p* = 0.006) was associated with superior OS and longer IFPFS (HR = 0.211; *p* = 0.029) in our study. BED_10_ ≥ 100 Gy has been proven to be correlated with superior local control [43,44]. This has also been corroborated in a large-scale SABR-based study by Rico et al. using the international RSSearch^®^ Patient Registry, which showed that BED_10_ ≥ 100 Gy treatment could improve local control in pulmonary oligometastases [43]. Other studies have documented various ranges of BED_10_ i.e., 48–105.6 Gy, revealing a two-year local control rate of 54–95% for pulmonary oligo-recurrence treated with SABR [17,18,19,20,29,34]. Whereas the three-year local control rates have been reported as 71.1% for lesions treated with BED_10_ ≥ 100 Gy and 44.9% for those treated with BED_10_ < 100 Gy. Consistent with relevant reports, the present study observed an improved two-year IFPFS of 74.2% which is associated with higher BED_10_. Our subset analysis revealed that the two-year IFPFS and OS for patients treated with BED_10_ ≥ 100 Gy (N = 27) were 85.1% and 69.6%, respectively, compared to 54.1% and 35.9% for patients treated with BED_10_ < 100 Gy (N = 18). Despite 42.2% (N = 19) of our patients having ≥2 lung tumors, an acceptable toxicity profile with 8.9% Grade 2–3 radiation pneumonitis was observed. 

We also examined the significant factors related to OFPFS for lung oligometastases after SABR treatment. Oligoprogressive status (HR = 2.396; *p* = 0.017) and EPD (HR = 2.376; *p* = 0.019) were found as unfavorable independent prognostic factors for OFPFS. Although few studies have investigated OFPFS after SABR, out-field failure has been observed as the major pattern of disease progression after SABR [45]. Notably, an inferior progression-free survival after SABR has been reported for synchronous metastases compared to metachronous metastases [46]. Our data revealed that DFI < 24 months, which included synchronous metastases (HR = 2.729; *p* = 0.041), was an independent prognostic factor for inferior OS. This result was compatible with previous SABR literature studies, which correlated improved survival with DFI ≥ 24 [18] 30 [20], 31.9 [17], and 36 months [29] in patients with pulmonary oligo-recurrence. However, our study did not find any significant association between synchronous metastasis and OFPFS, probably due to controlled EPD being one of our patient selection criteria. Nonetheless, the presence of EPD still represented a higher tumor burden, which might be attributable to a higher risk of out-field treatment failure.

The limitation of our study includes its retrospective design, relatively small sample size, and heterogeneous primary cancer types. We assumed an α/β ratio of 10 Gy for metastatic lung tumors as most cancer types in our study indicated low fractionation sensitivity. Though the α/β ratio of metastatic tumors at different sites is yet to be clarified, most studies indicate a consensus on the α/β ratio as 10 Gy [18,19,20,43]. To minimize the heterogeneous calculation of the biological tumor doses, this study applied an α/β ratio of 10 Gy for all metastatic lung tumors, which can be easily employed in clinical practice. With stringent patient selection criteria, we demonstrated the inferior treatment outcome of oligoprogressive compared with controlled status for lung metastases after SABR. With oligoprogression becoming increasingly common, prospective studies should be conducted to optimize patient selection for SABR and integrate individualized systemic therapy to reduce the risk of out-field treatment failure. 

## 5. Conclusions

Substantial in-field tumor control and longer OS could be achieved through SABR with BED_10_ ≥ 100 Gy for systemic therapy respondents with inoperable pulmonary oligometastases. However, oligoprogressive lung tumors demonstrated a higher risk of out-field treatment failure and inferior survival outcomes compared with controlled metastatic lung tumors. Thus, the metastatic tumor status after upfront systemic therapy should be taken into account to optimize patient selection of SABR for lung metastases. Subsequent systemic therapy should be tailored for patients with oligoprogression to reduce the risk of out-field treatment failure. However, in the absence of effective systemic therapy, SABR is a reasonable alternative to reduce resistant tumor burden.

## Figures and Tables

**Table 1:** Patient, tumor, and treatment characteristics (N = 45).

Characteristics	N = 45
Age, years, median (range)	60 (31–84)
Sex (%)	
Male	28 (62.2)
Female	17 (37.8)
ECOG performance status (%)	
0	24 (53.3)
1–2	21 (46.7)
Primary cancer (%)	
Hepatocellular carcinoma	16 (35.6)
Colorectal cancer	13 (28.9)
Cervical cancer	3 (6.7)
Endometrial cancer	2 (4.4)
Nasopharyngeal cancer	2 (4.4)
Non-small cell lung cancer	2 (4.4)
Urothelial carcinoma	2 (4.4)
Breast cancer	1 (2.2)
Esophageal cancer	1 (2.2)
Melanoma	1 (2.2)
Renal cell carcinoma	1 (2.2)
Chondrosarcoma	1 (2.2)
Metastatic lung tumor status (%)	
Oligoprogressive	16 (35.6)
Controlled	29 (64.4)
Metastatic status (%)	
Synchronous metastases	9 (20.0)
Metachronous metastases	36 (80.0)
Disease-free interval (%)	
<12 months	23 (51.1)
≥12–<24 months	6 (13.3)
≥24–<36 months	8 (17.7)
≥36 months	8 (17.7)
Extrapulmonary disease (%)	
Yes	19 (42.2)
No	26 (57.8)
Subsequent systemic therapy (%)	
Yes	30 (66.7)
No	15 (33.3)
Number of lung tumors (%)	
1	26 (57.8)
2–3	17 (37.8)
4–5	2 (4.4)
Tumor volume, mL, mean ± SD	4.45 ± 4.17
BED_10_, Gy, mean ± SD	93.36 ± 21.10

ECOG: Eastern Cooperative Oncology Group; SABR: Stereotactic Ablative Radiotherapy: BED_10_: Biologically effective dose, α/β = 10 Gy.

**Table 2:** Univariate analysis for OS, IFPFS, and OFPFS. OS, overall survival; IFPFS, in-field progression-free survival; OFPFS, out-field progression-free survival; ECOG, Eastern Cooperative Oncology Group; DFI, disease-free interval; BED_10_, biologically effective dose, α/β = 10 Gy, HR, hazard ratio; 95%-CI, 95% confidence interval. Categorical variables were sex, ECOG, primary cancer, metastatic lung tumor status (controlled vs. oligoprogressive), synchronous metastases (metachronous metastases ref.), disease-free interval (≥24 vs. <24 months), extrapulmonary disease (no vs. yes), subsequent systemic therapy (no vs. yes), and BED_10_ (<100 vs. ≥100 Gy), while the others were continuous variables. † Statistically significant.

Variables	OS			IFPFS			OFPFS		
	HR	95%-CI	*p*-Value	HR	95%-CI	*p*-Value	HR	95%-CI	*p*-Value
Age (<65 vs. ≥65 years)	0.861	[0.370; 2.001]	0.728	2.412	[0.647; 8.994]	0.190	0.637	[0.313; 1.296]	0.213
Sex (female vs. male)	1.441	[0.653; 3.179]	0.366	1.367	[0.342; 5.469]	0.659	1.246	[0.635; 2.446]	0.522
ECOG (0 vs. 1–2)	1.463	[0.652; 3.281]	0.356	0.701	[0.188; 2.610]	0.596	1.725	[0.884; 3.367]	0.110
Primary cancer	-	-	0.566	-	-	0.865	-	-	0.378
Metastatic lung tumor status	2.874	[1.235; 6.685]	0.014 †	0.824	[0.169; 4.010]	0.811	2.647	[1.322; 5.300]	0.006 †
Synchronous metastases	1.344	[0.565; 3.199]	0.504	1.122	[0.233; 5.410]	0.886	1.450	[0.672; 3.128]	0.343
DFI (≥24 vs. <24 months)	2.558	[1.016; 6.444]	0.046 †	0.511	[0.137; 1.916]	0.320	1.722	[0.857; 3.462]	0.127
Extrapulmonary disease	2.491	[1.101; 5.634]	0.028 †	0.536	[0.111; 2.596]	0.439	2.600	[1.297; 5.214]	0.007 †
Subsequent systemic therapy	1.677	[0.619; 4.543]	0.310	5.125	[0.637; 41.214]	0.124	1.403	[0.691; 2.849]	0.348
Number of lung tumors	1.255	[0.844; 1.867]	0.262	1.455	[0.725; 2.921]	0.292	0.913	[0.633; 1.318]	0.628
Tumor volume, mL	1.040	[0.942; 1.148]	0.437	1.088	[0.953; 1.243]	0.213	0.990	[0.914; 1.073]	0.808
BED_10_ (<100 vs. ≥100 Gy)	0.370	[0.165; 0.831]	0.016 †	0.211	[0.052; 0.851]	0.029 †	0.835	[0.427; 1.633]	0.598

**Table 3:** Multivariate analysis for OS, IFPFS, and OFPFS. OS, overall survival; IFPFS, in-field progression-free survival; OFPFS, out-field progression-free survival; ECOG, Eastern Cooperative Oncology Group; DFI, disease-free interval; BED_10_, biologically effective dose, α/β = 10 Gy, HR, hazard ratio; 95%-CI, 95% confidence interval. Metastatic lung tumor status (controlled vs. oligoprogressive), disease-free interval (≥24 vs. <24 months), extrapulmonary disease (no vs. yes), and BED_10_ (<100 vs. ≥100 Gy) were categorical variables. † Statistically significant.

Variables	OS			IFPFS			OFPFS		
	HR	95%-CI	*p*-Value	HR	95%-CI	*p*-Value	HR	95%-CI	*p*-Value
Metastatic lung tumor status	4.064	[1.605; 10.290]	0.003 †				2.396	[1.171; 4.901]	0.017 †
DFI (≥24 vs. <24 months)	2.729	[1.040; 7.161]	0.041 †						
Extrapulmonary disease							2.376	[1.154; 4.889]	0.019 †
BED_10_ (<100 vs. ≥100 Gy)	0.308	[0.134; 0.708]	0.006 †	0.211	[0.052; 0.851]	0.029 †			

## Data Availability

The data are available from the corresponding author upon reasonable request.

## Supplementary Materials

The following supporting information can be downloaded at: https://www.mdpi.com/article/10.3390/diagnostics13091597/s1, Supplementary Materials S1: Maximum dose constraints for 3–5 fractions SABR regimen.
