# Supplementary material for: Effectiveness of Stereotactic Ablative Radiotherapy for Systemic Therapy Respondents with Inoperable Pulmonary Oligometastases and Oligoprogression
Source: Diagnostics (Basel). 2023 Apr 29;13(9):1597. doi: 10.3390/diagnostics13091597 (PMC10177978; doi:10.3390/diagnostics13091597)
Supplement: Supplementary file 1 [file diagnostics-13-01597-s001.zip › diagnostics-2303947-supplementary.pdf]

Supplementary Materials S1

Maximum dose constraints for 3–5 fractions SABR regimen

**Organs at risk**	**3 fractions**	**4 fractions**	**5 fractions**
Brachial plexus	24 Gy (8 Gy/fx)	27.2 Gy (6.8 Gy/fx)	30.5 Gy (6.1 Gy/fx)
Esophagus	25.2 Gy (8.4 Gy/fx)	30 Gy (7.5 Gy/fx)	35 Gy (7 Gy/fx)
Heart/pericardium	30 Gy (10 Gy/fx)	34 Gy (8.5 Gy/fx)	38 Gy (7.6 Gy/fx)
Trachea and large bronchus	30 Gy (10 Gy/fx)	34.8 Gy (8.7 Gy/fx)	40 Gy (8 Gy/fx)
Rib	30 Gy (10 Gy/fx)	40 Gy (10 Gy/fx)	43 Gy (8.6 Gy/fx)
Spinal cord	18 Gy (6 Gy/fx)	26 Gy (6.5 Gy/fx)	30 Gy (6 Gy/fx)
Skin	30 Gy (10 Gy/fx)	36 Gy (9 Gy/fx)	32 Gy (6.4 Gy/fx)
Stomach	22.2 Gy (7.4 Gy/fx)	27.2 Gy (6.8 Gy/fx)	32 Gy (6.4 Gy/fx)
